# Myocardial function in aortic stenosis – insights from radial multilayer Doppler strain

**DOI:** 10.1186/s12947-015-0001-z

**Published:** 2015-02-19

**Authors:** Dana Cramariuc, Eva Gerdts, Johannes Just Hjertaas, Alexandru Cramariuc, Einar Skulstad Davidsen, Knut Matre

**Affiliations:** Department of Heart Disease, Haukeland University Hospital, Bergen, Norway; Department of Clinical Science, University of Bergen, Bergen, Norway

**Keywords:** Aortic stenosis, Hypertension, Radial strain, Myocardial function, Tissue Doppler imaging

## Abstract

**Background:**

Left ventricular (LV) radial tissue Doppler imaging (TDI) strain increases gradually from the subepicardial to the subendocardial layer in healthy individuals. A speckle tracking echocardiography study suggested this gradient to be reduced in parallel with increasing aortic stenosis (AS) severity.

**Methods:**

We used TDI strain in 84 patients with AS (mean age 73 ± 10 years, 56% hypertensive) for superior assessment of layer strain. 38 patients had non-severe and 46 severe AS by aortic valve area corrected for pressure recovery. Peak systolic radial TDI strain was measured in the subendocardial, mid-myocardial and subepicardial layers of the basal inferior LV wall, each within a region of interest of 2 × 6 mm (strain length 2 mm).

**Results:**

Radial strain was lower in the subepicardial layer (33.4 ± 38.6%) compared to the mid-myocardial and subendocardial layers (50.3 ± 37.3% and 53.0 ± 40.0%, respectively, both p < 0.001 vs. subepicardial). In the subendo- and midmyocardium, radial strain was lower in patients with severe AS compared to those with non-severe AS (p < 0.05). In multivariate regression analyses including age, heart rate, inferior wall thickness, hypertension, and AS severity, radial strain in the mid-myocardium was primarily attenuated by presence of hypertension (β = −0.23) and AS severity (β = −0.26, both p < 0.05), while radial strain in the subendocardium was significantly influenced by AS severity only (β = −0.35, p < 0.01).

**Conclusions:**

In AS, both the AS severity and concomitant hypertension attenuate radial TDI strain in the inferior LV wall. The subendocardial radial strain is mainly influenced by AS severity, while midmyocardial radial strain is attenuated by both hypertension and AS severity.

## Background

Several publications demonstrating the feasibility of assessing strain in multiple myocardial layers in experimental [1-4] and clinical [5-11] settings have suggested that disease might affect only parts of the myocardial wall or have a differential effect on individual myocardial layers. A gradual increase in strain from the subepicardial to the subendocardial layer in healthy myocardium has been demonstrated in previous studies [1,7]. Recently it has been shown that multilayer measurement of strain can be used for assessment of myocardial ischemia [12,13].

In patients with aortic stenosis (AS), left ventricular (LV) hypertrophy and concentric geometry develop during progression of the AS [14], and LV global systolic function by either ejection fraction, midwall shortening or global LV longitudinal strain decreases in advanced stages of disease [15,16]. Recently, reduced endocardial to epicardial radial speckle strain ratio was reported in severe AS [17]. More detailed information about determinants of change in regional function during progression of AS may be obtained by integrating clinical information and assessment of tissue Doppler imaging (TDI) strain in multiple myocardial layers, a method shown to be superior to speckle tracking for detection of layer dysfunction in an experimental study on ischemia [2]. Thus TDI radial strain was chosen for the present study to assess the overall deformation of differentially orientated myocardial fibers within the individual virtual endocardial, midwall and epicardial myocardial layers in patients with AS and its relation to disease severity and other clinical covariates.

## Methods

### Study population

The present mechanistic study was prospectively planned for all patients with calcific AS who had conventional and TDI echocardiography undertaken at the Echocardiography laboratory, Haukeland University Hospital, Bergen, Norway as part of prospective clinical trial protocols in the time period March 2006-August 2008. A total of 84 patients were identified, and all accepted participation in the study. Thirty-eight patients had initially non-severe AS and came for scheduled study echocardiograms in the Simvastatin Ezetimibe in Aortic Stenosis (SEAS) study [18]. Forty-six patients with severe AS were recruited consecutively in the same period at the screening visit for a multicenter trial comparing the benefits of Mosaic Ultra vs. Perimount Magna aortic supraannular bioprostheses in AS [19]. Patients with a history of myocardial infarction or percutaneous coronary revascularization, cerebrovascular disease, more than mild mitral or aortic regurgitation, atrial arrhythmias, as well as patients with a suspicion of primary hypertrophic cardiomyopathy were excluded from the current analysis.

Coronary angiography was performed preoperatively in patients with severe symptomatic AS.

History of hypertension was defined as known hypertension reported by the attending physician or antihypertensive treatment at the examination date. All patients gave written informed consent to participate in the study, which was approved by the regional Ethics Committees.

### Conventional echocardiography

All examinations were performed using a Vivid 7 echocardiograph (GE Vingmed Ultrasound, Horten, Norway) equipped with a phased-array transducer following the previously published SEAS protocol [20,21]. Postprocessing was performed on dedicated workstations using stored digital loops of 5 cardiac cycles with ECG display and recording of impedance-derived respiration.

#### LV geometry

LV dimensions were measured in parasternal long-axis view according to current guidelines [22]. LV hypertrophy was assessed by LV mass/height^2.7^ using gender-specific prognostically validated cut-off values (cut-offs 46.7 g/m^2.7^/49.2 g/m^2.7^ in women/men) [21]. Relative wall thickness was calculated from posterior LV wall thickness/LV internal radius ratio at end-diastole and considered increased if ≥0.43. LV geometry was assessed from LV mass index and relative wall thickness in combination: patients with normal LV mass were divided into normal LV geometry and concentric remodeling groups, and patients with LV hypertrophy into eccentric and concentric hypertrophy groups [23].

#### LV systolic function

LV endocardial systolic function was assessed by biplane Simpson’s ejection fraction (low if <50%) [22]. Circumferential end-systolic stress was calculated at midwall using a cylindrical model, and peak systolic stress was estimated from systolic blood pressure and end-diastolic LV dimensions taking into account the mean aortic valve gradient and using an invasively validated equation [24].

#### AS severity

Doppler assessment of AS included measurement of peak and mean transvalvular velocities and gradients, as recommended by the European Society of Cardiology guidelines [25]. To avoid overestimation of disease severity in patients with less severe AS, also pressure recovery adjusted aortic valve area (i.e. energy loss index [ELI]) was calculated [25-27]. Severe AS was defined as ELI <0.60 cm^2^/m^2^ [26].

### Tissue Doppler imaging

**Figure 1:**
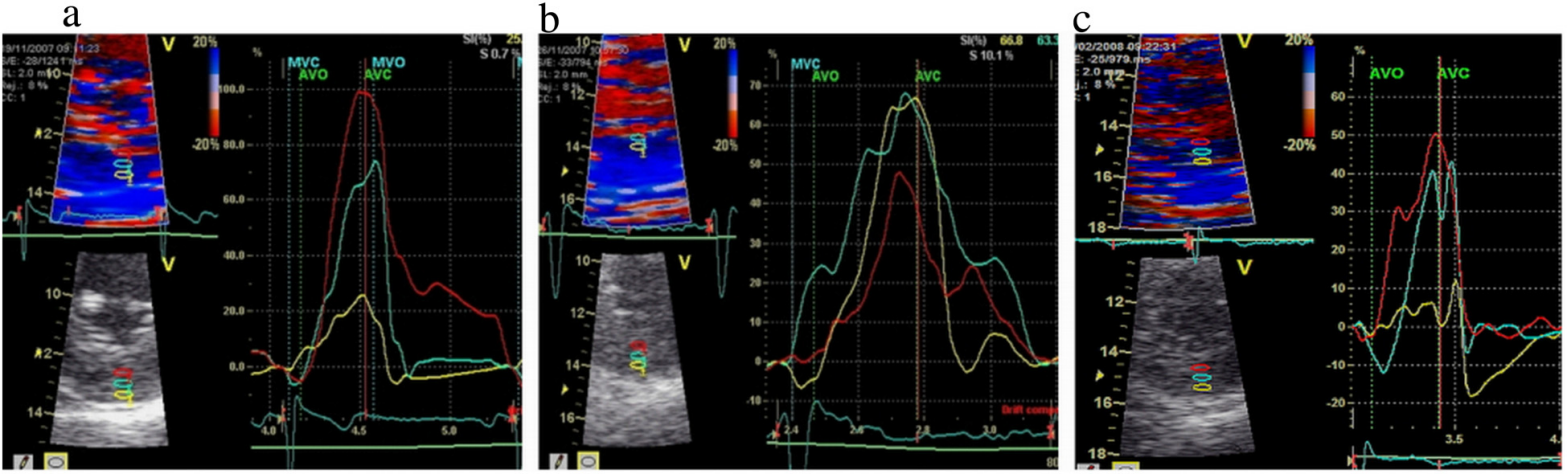
**Measurement of peak systolic radial strain in three layers in the left ventricular inferior wall of: 1a) one normotensive patient with non-severe aortic stenosis; 1b) one normotensive patient with severe aortic stenosis; 1c) one hypertensive patient with severe aortic stenosis.** Each panel: Top left – Colour TDI image in parasternal short-axis view of the inferior left ventricular wall. Bottom left – B-mode image with 3 regions of interest placed in three layers (subendocardium, mid-myocardium, subepicardium) in the left ventricular inferior wall. Right panel: corresponding peak systolic radial strain curves for the three regions of interest: red curve for subendocardium, blue curve for mid-myocardium, and yellow curve for subepicardium.

TDI recordings were analyzed for strain in the subendocardial, midmyocardial and subepicardial layers of the inferior LV wall using high resolution-zoomed parasternal short-axis recordings (at the level of papillary muscles) (Figure 1), using our highly reproducible method reported in healthy subjects [7]. The inferior wall was chosen to avoid angle-induced errors in TDI-analyses and noise due to reverberations. Lateral averaging was set at maximum and radial averaging at minimum for better deformation sampling of the myocardial layers. The frame rate varied between 225 and 327/s for TDI acquisitions.

Image post-processing was performed using EchoPac version BT 09 (GE Vingmed Ultrasound, Horten, Norway). By anatomical M-mode through the area to be analyzed, we selected one cardiac cycle with best quality in terms of lack of drop-outs and static artifacts. Three regions of interest of 2 mm height and 6 mm width were placed in the subendocardial, midmyocardial, and subepicardial layer of the inferior wall, and tracked during the cardiac cycle in order to assure an even distribution of the regions of interest at all times. Particular attention was given to correct placement in the subendocardial region in order to avoid sampling in the trabeculae or blood. Strain length was set at 2 mm to minimize any overlap between areas during strain analyses. Aortic valve closure was identified from a pulsed wave Doppler recording in the LV outflow tract. Peak systolic radial strain was measured in the subendocardial (EndoS), midmyocardial (MidS), and subepicardial layer (EpiS). The inter- and intraobserver variability for this method was excellent (intraclass correlation coefficient 0.94 and 0.98, respectively) as previously published in a separate study from our experimental laboratory [2].

### Statistical analysis

Data management and analysis were performed using IBM SPSS Statistics 19.0 (SPSS, Chicago, IL) software. Data are presented as mean ± standard deviation for continuous variables and as percentages for categorical variables. The *χ*^2^ test was used to compare categorical variables and full-factorial two-way ANOVA with Sidak’s post hoc test to compare continuous variables, as appropriate. Univariate correlates of strain were identified by Pearson’s correlation for normally distributed data. Predictors of higher peak strain (EndoS and MidS) were assessed in two multiple linear regression models with collinearity diagnostics. Results are presented as multiple R^2^ for the model and ß-coefficients for significant covariates. Both EndoS and MidS were then dichotomized according to the median value. The discrimination power of the multivariate models was checked using C-statistics for linear regressions by plotting the standardized predictive values against high EndoS and MidS, respectively, in Receiver Operator Characteristics (ROC) analyses. Two-tailed p < 0.05 was considered significant both in univariate and multivariate analyses.

## Results

### Baseline patient characteristics

All patients had normal regional wall motion of the inferior wall, and ejection fraction was normal in 80 patients (95%) and mildly reduced (between 40 and 50%) in 4 patients. Patients with severe AS were on average older and had higher prevalences of LV hypertrophy and concentric LV geometry, compared to patients with non-severe AS, while blood pressure and heart rate did not differ between groups (Table 1).

**Table 1:** **Baseline characteristics of the total study population and in subgroups of non-severe vs. severe AS**

	**All (n = 84)**	**Non-severe AS (n = 38)**	**Severe AS (n = 46)**
Age (years)	73 ± 10	70 ± 10	76 ± 8*
Women	56%	50%	61%
Body mass index (kg/m^2^)	25.2 ± 4.0	25.4 ± 3.8	25.0 ± 4.2
Heart rate (beats /minute)	65 ± 10	64 ± 9	66 ± 10
Hypertension	56%	61%	52%
Systolic blood pressure (mm Hg)	149 ± 20	148 ± 17	149 ± 23
Diastolic blood pressure (mm Hg)	79 ± 12	81 ± 11	77 ± 12
Ejection fraction (%)	63 ± 7	64 ± 6	62 ± 8
Circumferential end-systolic stress (dyne/cm^2^)	135 ± 44	134 ± 43	135 ± 45
Peak systolic stress (dyne/cm^2^)	242 ± 74	253 ± 83	234 ± 66
LV mass (g)	217 ± 73	206 ± 74	226 ± 71
LV hypertrophy	57%	45%	67%‡
Concentric LV geometry	60%	42%	74%†
Aortic valve area index (cm^2^/m^2^)	0.53 ± 0.20	0.69 ± 0.17	0.40 ± 0.09*
ELI (cm^2^/m^2^)	0.60 ± 0.25	0.81 ± 0.22	0.44 ± 0.10*
EndoS (%)	53.0 ± 40.0	70.5 ± 37.6	38.4 ± 36.2*
MidS (%)	50.3 ± 37.3	60.1 ± 43.1	42.1 ± 29.7‡
EpiS (%)	33.4 ± 38.6	41.5 ± 47.4	26.7 ± 28.1

Data are mean ± SD or percentage. * p <0.001, † p <0.01, and ‡ p <0.05. LV = left ventricular; ELI = energy loss index; EndoS, MidS and EpiS = peak systolic radial strain in the subendocardial, midmyocardial, and subepicardial layers of the left ventricular inferior wall.

### Covariates of radial strain in the basal inferior LV wall

#### -Relation to degree of AS

**Figure 2:**
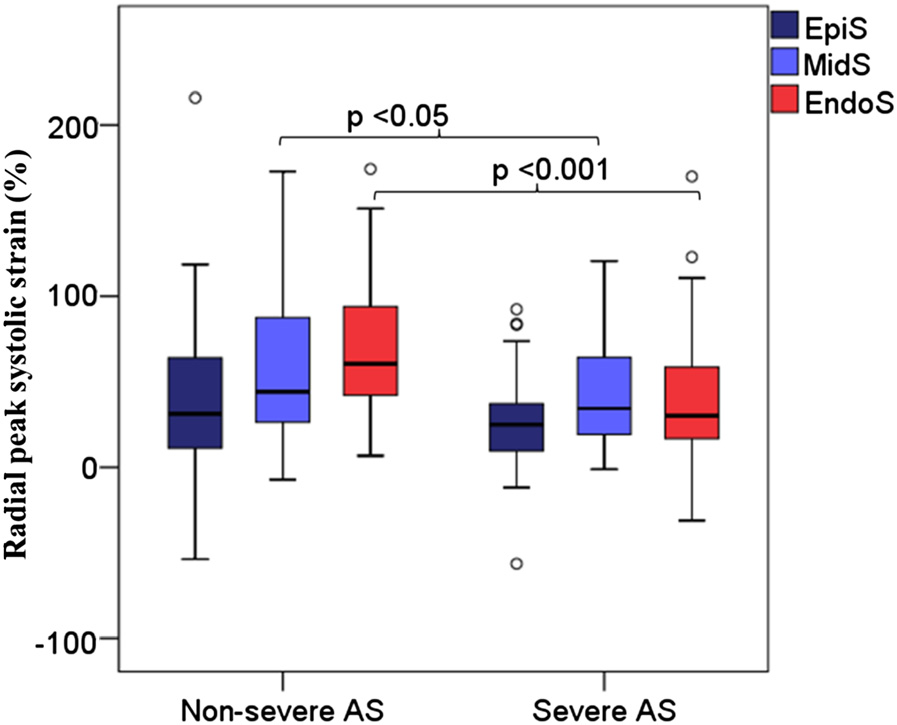
**Comparison of peak systolic radial strain (vertical axis) in three myocardial layers (subendocardium: EndoS, mid-myocardium: MidS, and subepicardium: EpiS) between patients with non-severe vs. severe aortic stenosis (AS) (horizontal axis).** P <0.001 for comparison of EndoS and p <0.05 for MidS between the two groups, respectively.

In the total study population, EndoS and MidS had comparable values, while S was significantly lower in the subepicardium (p < 0.001, Table 1). Grouping patients according to severity of AS, EndoS and MidS were significantly lower in patients with severe vs. non-severe AS (Figure 2).

Among patients with symptomatic AS undergoing coronary angiography, 50% had significant coronary artery disease requiring revascularization by coronary bypass grafting. This subgroup of patients had numerically, but not statistically significant, lower MidS (44.1 ± 28.7% vs. 52.9 ± 40.3%, p = 0.26) and EndoS (43.0 ± 39.2% vs. 57.2 ± 39.8%, p = 0.14).

### -Relation to LV geometry

**Figure 3:**
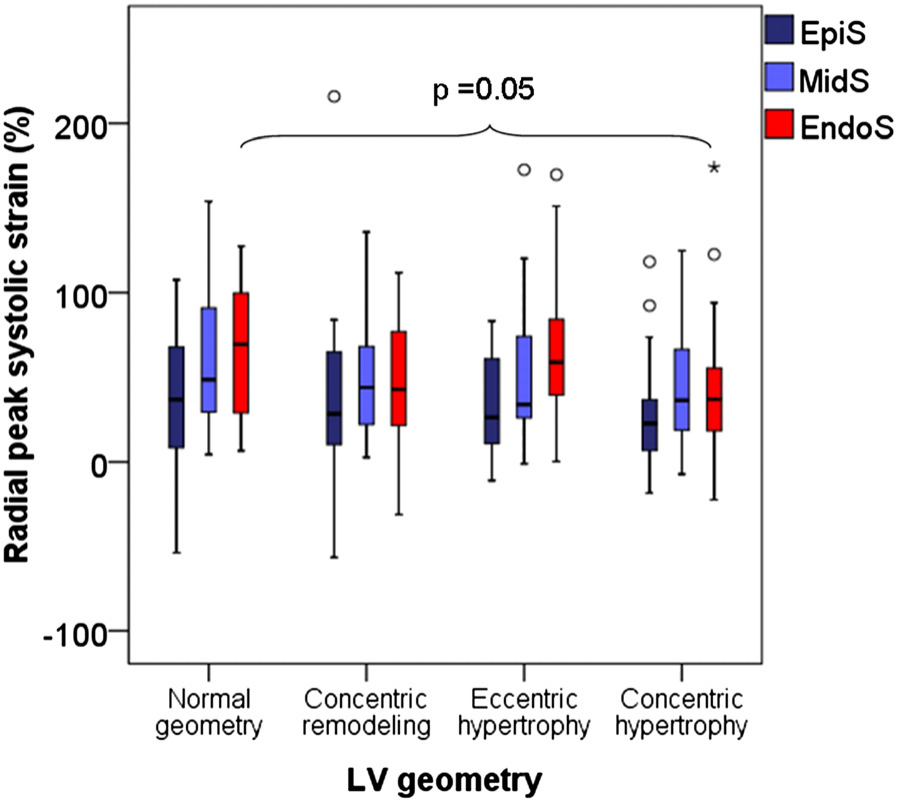
**Comparison of peak radial systolic strain (vertical axis) in three myocardial layers (subendocardium: EndoS, mid-myocardium: MidS, and subepicardium: EpiS) in the four left ventricular (LV) geometric groups (horizontal axis).** P <0.05 for comparison of EndoS between patients with normal LV geometry vs. concentric LV hypertrophy.

EndoS was significantly lower in patients with concentric LV geometry: 43.8 ± 36.1 vs. 66.4 ± 42.0 (p < 0.05). When compared between the four LV geometric groups, EndoS was lowest in patients with concentric LV hypertrophy, and highest in those with normal LV geometry (Figure 3). MidS and EpiS did not differ significantly between different LV geometric groups (Figure 3).

### -Relation to hypertension

MidS tended to be lower in patients with hypertension: 43.7 ± 38.3 vs. 58.6 ± 34.6, p = 0.06, while EpiS and EndoS were similar for these groups.

### -Relation to wall stress

**Figure 4:**
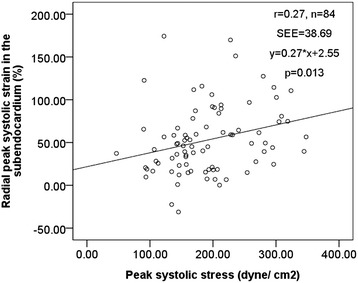
**The relation between subendocardial peak systolic radial strain (vertical axis) and peak systolic stress (horizontal axis).** Pearson correlation coefficient r = 0.21, p = 0.05.

EndoS increased with increasing peak wall systolic stress in univariate analyses (r = 0.21, p = 0.05) (Figure 4). Peak wall systolic stress was negatively correlated with presence of concentric LV geometry (r = −0.78) and higher LV mass (r = −0.22), both p < 0.05.

### -Multivariate analyses

In multivariate regression models including age, heart rate, the end-diastolic thickness of the LV inferior wall, and indicator variables for severity of AS and history of hypertension, lower EndoS was associated with presence of severe AS (β = −0.35, p < 0.01), while lower MidS was associated with severe AS and hypertension (β = −0.26 and −0.23, respectively, both p = 0.03) independent of the other covariates. Further including EpiS in the models did not change the results for EndoS, while lower MidS was no longer associated with severity of AS, but significantly predicted by hypertension (Table 2). Gender, presence of significant coronary artery disease, blood pressure, and systemic arterial compliance, were not significant predictors of EndoS and MidS when alternatively introduced in further multivariate analyses. Replacing end-diastolic thickness of the inferior LV wall with LV mass in the models did not change the results. None of the multivariate models were significant when applied to EpiS only.

**Table 2:** **Predictors of EndoS and separately MidS in multivariate regression analyses**

**Dependent variables**	**EndoS (multiple R** ^**2**^ **= 0.22, p <0.01)**		**MidS (multiple R** ^**2**^ **= 0.39, p <0.001)**	
	**ß**	**P**	**ß**	**p**
Age (yrs)	−0.11	0.33	0.05	0.60
Hypertension	−0.10	0.35	−0.25	0.01
Heart rate (beats/minute)	−0.08	0.44	−0.18	0.06
Inferior LV wall thickness (cm)	−0.08	0.49	−0.08	0.42
Severe AS by ELI	−0.33	<0.01	−0.14	0.17
EpiS (%)	0.08	0.43	0.52	<0.01

LV = left ventricular; ELI = energy loss index; EpiS, MidS and EndoS = peak systolic radial strain in the subepicardial, midmyocardial, and subendocardial layer of the left ventricular inferior wall.

Variables that did not enter any of the models: gender, coronary artery disease requiring revascularization, systolic blood pressure, diastolic blood pressure, systemic arterial compliance.

## Discussion

Few studies have reported on myocardial function by multilayer strain in AS [10,17]. As previously reported, the myocardial systolic deformation is the result of the interplay between the three myocardial layers: the subendocardial and subepicardial layers, which are longitudinally aligned fibers, and the midmyocardial layer with circumferentially aligned fibers. This deformation including cross-fiber shortening results in thickening measured as radial strain that exceeds the fiber shortening. Patients with AS have progressive changes in left ventricular geometry achieving higher prevalence of left ventricular hypertrophy and concentric left ventricular geometry in the severe stage [14]. A change in layer geometry and also in the functional interaction that determines the regional radial strain is thus to be expected. Adding to previous knowledge, this study demonstrates that in patients with AS, radial TDI strain is reduced both in the endocardial and midmyocardial layers of the LV inferior wall, but more markedly in the subendocardium, resulting in disappearance of the normal radial strain gradient (i.e. a gradual increase in strain from the subepicardium to the subendocardium) found in healthy individuals [7]. As demonstrated, radial TDI strain in the subendocardium primarily reflects AS severity and associated changes in LV geometry and wall stress. In contrast, radial TDI midwall strain is mostly affected by history of hypertension, while no significant covariate of radial subepicardial strain was identified.

### TDI in myocardial layers in AS

TDI assessment of myocardial function in small regions of the LV wall can be carried out by using carefully selected dimensions of the regions of interest and strain length based on previous experience [1,7]. The reproducibility of our method has previously been demonstrated in healthy individuals [7], while its advantages in detecting layer dysfunction compared to speckle tracking is known from experimental studies on ischemia [2].

By using TDI, we demonstrate in the present study that multilayer radial strain in the inferior LV wall is reduced progressively with increasing AS severity and wall stress, and most markedly in the subendocardium, which is the myocardial layer responsible for most of the long axis shortening during systole. This functional loss may be irreversible in advanced AS. Recently, reduced radial subendocardial to subepicardial strain ratio was reported from a study in 73 patients with AS using velocity vector imaging analysis of two layers (outer and inner halves) in the LV wall [17]. The present study adds to these findings by reporting deformation measurements by TDI, a method that avoids spatial averaging over myocardial areas with possible overlap between myocardial layers, and thus avoids underestimation of the strain gradient [2].

As suggested by previous clinical studies of LV geometry in chronic pressure overload conditions [15,28], as well as theoretical models of LV mechanics [29], pressure overload due to AS has a differential effect on myocardial layer function depending on fiber orientation. Radial strain in the subendocardial layer, where most fibers are in the longitudinal direction, was in the present study most sensitive to an increased AS severity. This confirms our previous finding of lower longitudinal strain in AS with altered LV geometry [16]. The particularly negative impact of the progression of AS on subendocardial radial strain may reflect the increasing mismatch between subendocardial blood flow and oxygen demand as a result of increasing wall thickness. It might also reflect altered tissue structure with more subendocardial fibrosis when the wall is concentrically hypertrophied [30]. In this context, the positive relationship between peak systolic radial strain in the endocardium and peak systolic stress might appear paradoxical. However, deformation measured as radial strain is the summation of the shortening and thickening of the cardiomyocytes in the three layers creating a contraction force oriented towards the center of the left ventricle [31]. Consequently, radial strain increases from the subepicardium (where thickening of longitudinally oriented fibers occurs) to the midmyocardium (due to thickening and shortening of the circumferential fibers) and further to the subendocardium (where thickening of the subendocardial longitudinal fibers adds up). At the same time, the subendocardial layer is receiving the highest tension during the increase in systolic pressure, with lower wall stress in the other two layers, resulting in the observed positive relation between peak systolic strain and peak systolic stress. Of note, the observed increase in peak systolic strain from the subepicardial to the subendocardial layer in the present study population was less than that previously reported in healthy hearts [7].

Hypertension is highly prevalent among older patients with AS [21,32] and associated with more advanced LV hypertrophy and higher mortality and ischemic CV events even in mild-moderate AS [14]. Hypertension is often associated with depressed midwall function despite normal ejection fraction [33]. In a more recent study using both cardiac magnetic resonance imaging (MRI) and echocardiography in hypertensive patients, a marked reduction in circumferential strain across the wall was shown by MRI in patients with LV hypertrophy [34]. As demonstrated, the midmyocardial layer of the LV wall, mostly with circumferential fibers, was primarily influenced by hypertension in our study population. These results reflect that concentric LV geometry and in particular concentric LV hypertrophy, the most common type of LV adaptation in severe AS, is associated with micro- and macrovascular dysfunction which in turn leads to ischemia-induced fibrosis and myocardial dysfunction, particularly in the midmyocardial layer [35].

Radial strain in the subepicardial layer was less influenced by the clinical and echocardiographic factors recorded in the present study. However, EpiS values were significantly lower than previously reported in healthy individuals [7].

### Study limitations

The present study focuses on regional changes in peak systolic radial strain in three virtual myocardial layers in a segment of the posterior LV wall in patients with aortic stenosis. This is one of several measures of regional deformation, and should not be interpreted as a measure of global myocardial function.

The accuracy of TDI strain analysis depends highly on image quality and angle. All patients included in the present study had a high-quality, zoomed parasternal short-axis image of the LV inferior wall. All acquisitions had high temporal resolution. Moreover, a high prevalence of LV hypertrophy and concentric geometry in this population allowed good layer separation.

Coronary artery disease was assessed by preoperative angiography only in symptomatic patients with scheduled aortic valve replacement. Presence of subclinical coronary artery disease cannot be excluded in the other patients; however, only critical coronary artery stenosis would impact resting myocardial function [36]. No patients had a history of myocardial infarction or regional motion abnormality on the echocardiogram.

Because of the angle dependency of TDI, perpendicular segments to the ultrasound beam like the inferior and anterior wall are to be preferred. We chose the inferior wall for the present analyses in order to avoid angle errors and possible reverberation artifacts in the near-field of the transducer as well as sampling in the septum. Strain is heterogeneous in the LV wall [34]. However, as previously reported by MRI, an even stronger impact of hypertension and aortic stenosis on myocardial strain might be expected in the anterior wall [34].

## Conclusions

Severe AS was accompanied by reduction of radial TDI strain in the midmyocardial and the subendocardial layers of the basal inferior LV wall. The radial subendocardial strain was mostly affected by AS severity and associated changes in LV geometry and wall stress. The radial midmyocardial strain was primarily influenced by hypertension, but also by severity of AS.

## Abbreviations

AS: Aortic valve stenosis
ELI: Energy loss index
EndoS: Peak radial systolic strain in the subendocardium
EpiS: Peak radial systolic strain in the subepicardium
LV: Left ventricle
MidS: Peak radial systolic strain in the midmyocardium
TDI: Tissue Doppler imaging
